# The mitochondrial genome of *Epiphragma* (*Epiphragma*) *mediale* (Diptera: Limoniidae)

**DOI:** 10.1080/23802359.2021.1907808

**Published:** 2021-03-31

**Authors:** Bing Zhang, Shang Gao, Ding Yang

**Affiliations:** Department of Entomology, College of Plant Protection, China Agricultural University, Beijing, China

**Keywords:** Mitochondrial genome, Limnophilinae, Phylogenetics

## Abstract

The crane fly *Epiphragma* (*Epiphragma*) *mediale* Mao and Yang, belongs to subfamily Limnophilinae of family Limoniidae. This mitogenome of *E.* (*E.*) *mediale* was sequenced used next-generation sequencing (NGS), the new representative of the mitogenome of the subfamily. The nearly complete mitogenome is 14,858 bp totally, consisting of 13 protein-coding genes, 2 rRNAs and 22 transfer RNAs. All genes have the similar locations and strands with that of other published species of Limoniidae. The nucleotide composition biases toward A and T, which together made up 75.2% of the entirety. Bayesian inference analysis strongly supported the monophyly of Tipuloidea. It suggested that Pediciidae is the basal clade of Tipuloidea and the monophyletic Tipulidae was assigned as the sister to the monophyletic Cylindrotomidae. The phylogenetic relationship within Tipuloidea was as follows: Pediciidae + (Limoniidae + (Tipulidae + Cylindrotomidae)).

## Introduction

The crane flies are one of the largest groups of the suborder Nematocera (Diptera), including over 15,000 extant species in approximately 500 genera and subgenera worldwide (Oosterbroek 2021). Adults of this group are commonly found in moist forested areas near ponds and streams, but also occur in grasslands, cultivated fields, urban yards and even deserts. The immature stages of most species live in aquatic or semi aquatic habitats, but many species with terrestrial larvae are also known (Alexander and Byers 1981; Gelhaus 2005; De et al. 2008; Ribeiro 2008). The subgenus *Epiphragma* (*Epiphragma*) Osten Sacken, 1860 is the largest subgenus in the genus *Epiphragma*, with 119 known species, and is considered to be monophyletic (Ribeiro 2008).

The specimens of *Epiphragma* (*Epiphragma*) *mediale* (Voucher number: CAUYDZBTTLLEE-Medi-1) used for this study were collected in Gongshan County (27°52′ N, 98°21′ E) of Yunnan Province by Bing Zhang on 8 May 2018, and then identified by Bing Zhang through the morphological character (Spur at origin of *Rs* almost obsolete; apical half of interbase like a slender rod bent at a 90-degree angle from thickened base) (Mao and Yang 2009). Specimens are deposited in the Entomological Museum of China Agricultural University (CAU). The total genomic DNA was extracted from the whole body (except head, legs and wings) of the specimen using the DNeasy Blood & Tissue Kit (Qiagen, Germany) and stored at －20 °C until needed. The mitogenome was sequenced used next generation sequencing (NGS) by Beijing Berry Genomics Co., Ltd. Library preparation and next generation sequencing (NGS) were conducted at Illumina Nova6000 platform and library size is 350 bp DNA reads and finally got 6 G sequencing data. We used the IDBA-UD (Peng et al. 2012) to assemble mtgenome from the NGS reads. This newly assembled mtgenome was first uploaded as raw fasta files to MITOS (Bernt et al. 2013) to identify open reading frames (ORFs), rRNAs and tRNAs, and each protein-coding gene were manually identified in MEGA 7.0 (Kumar et al. 2016) by aligned with others crane flies species.

For the Bayesian analyses, We aligned based on the conservation of reading frames by first translating into amino acids and aligning individually in MEGA 7.0 (Kumar et al. 2016), All these individual alignments were concatenated into a single matrix using Phylosuite (Zhang et al. 2020), we then used PartitionFinder2 (Lanfear et al. 2016) to search for the best-fit scheme as well as to estimate the model of nucleotide evolution for each partition using the Bayesian Information Criterion (BIC). we used the best-fit partitioning scheme and partition-specific models recommended by PartitionFinder2 (Lanfear et al. 2016) and analyzed using MrBayes 3.2.7a (Ronquist et al. 2012) on CIPRES (Miller et al. 2011).

The nearly complete mitogenome of *Epiphragma* (*Epiphragma*) *mediale* (Genbank accession number: MW368866) is 14,858 bp. It encoded 13 protein-coding genes (PCGs), 22 tRNA genes and two rRNA genes, but the control region could not be sequenced entirely in this study, and were similar with related reports before (Kang et al. 2019; Ren et al. 2019a, 2019b, 2019c; Zhang et al. 2019a, 2019b). All genes have the similar locations and strands with that of other published Limoniidae species. The nucleotide composition of the mitogenome was biased toward A and T, with 75.2% of A + T content (A = 38.1%, T = 37.1%, C = 15.0%, G = 9.8%). The A + T content of PCGs, tRNAs, and rRNAs is 74.0%, 77.5%, and 78.6%, respectively. The total length of all 13 mitochondrial PCGs of *E.* (*E.*) *mediale* is 11,211 bp. Two PCGs (*NAD2* and *NAD6*) initiate with ATT codons, and eight PCGs (*COI*, *COII*, *COIII*, *ATP6*, *NAD4, NAD4L, CYTB* and *NAD1*) initiate with ATG codons, *ATP8* and *NAD3* initiate with ATC as a start codon, *NAD5* initiates with GTG as a start codon, respectively. Twelve PCGs use the typical termination codons TAA, only one PCG (*NAD1*) use TAG in *E.* (*E.*) *mediale.*

**Figure 1.**
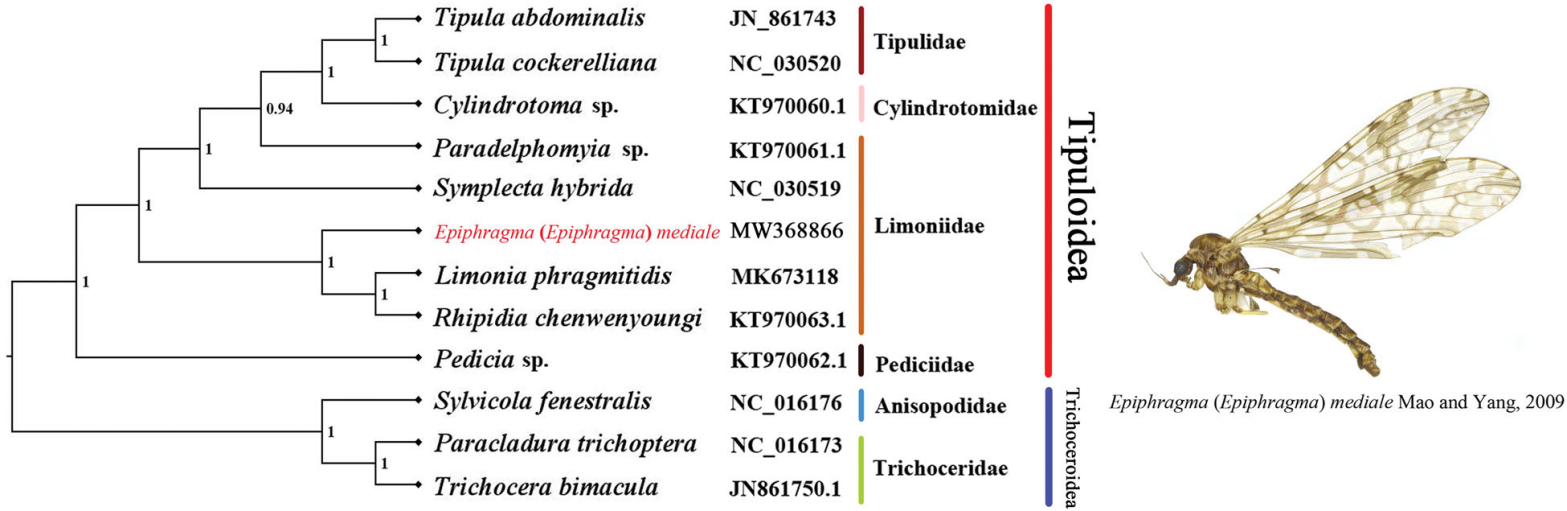
Bayesian phylogenetic tree of 12 Diptera species. The posterior probabilities are labeled at each node. Genbank accession numbers of all sequence used in the phylogenetic tree have been included in the figure and corresponding to the names of all species.

Phylogenetic analysis was performed based on the nucleotide sequences of 13 PCGs from 12 Diptera species. The phylogenetic tree topology was generated with Bayesian inference (BI) based on the PCGs matrices (Figure 1). According to the phylogenetic result, the monophyly of superfamily Tipuloidea was supported strongly. Pediciidae is the basal clade of Tipuloidea. The monophyletic Tipulidae was supported as the sister group to the monophyletic Cylindrotomidae. The phylogenetic relationship inferred from the Bayesian analysis in this text is very clear: Pediciidae + (Limoniidae + (Tipulidae + Cylindrotomidae)). The relationship among families within Tipuloidea was consistent with the previous study (Petersen et al. 2010). The mitogenome of *E.* (*E.*) *mediale* could not only provide the important information for the further studies of Tipuloidea phylogeny but also contribute to the taxonomic studies.

## Data Availability

Mitogenome data supporting this study are openly available in GenBank at https://www.ncbi.nlm.nih.gov/nuccore/MW368866, Associated BioProject, https://www.ncbi.nlm.nih.gov/bioproject/PRJNA706542, BioSample accession number at https://www.ncbi.nlm.nih.gov/biosample/SAMN18137747 and Sequence Read Archive at https://www.ncbi.nlm.nih.gov/sra/SRR13856859.
